# Characterization of soil microbes associated with a grazing‐tolerant grass species, *Stipa breviflora*, in the Inner Mongolian desert steppe

**DOI:** 10.1002/ece3.6715

**Published:** 2020-09-14

**Authors:** Lingling Chen, Yala Saixi, Ru Yi, Taogetao Baoyin

**Affiliations:** ^1^ Ministry of Education Key Laboratory of Ecology and Resource Use of the Mongolian Plateau & Inner Mongolia Key Laboratory of Grassland Ecology School of Ecology and Environment Inner Mongolia University Hohhot China

**Keywords:** bulk soil, co‐occurrence networks, grazing‐tolerant, rhizosphere, soil microbial community, *Stipa breviflora*

## Abstract

Although soil microbial communities are central in ecosystem functioning, we know little of their characterization for those associated with grazing‐tolerant host plant species in grassland ecosystems in response to grazing. In this study, we used a high‐throughput sequencing approach to characterize soil microbes from the rhizosphere and bulk soil of grazing‐tolerant grass species, *Stipa breviflora*, in the Inner Mongolian desert steppe. We found that response mechanisms of soil bacteria distinct from fungal communities, and variance also occur between the rhizosphere and bulk soil communities under long‐term grazing. Soil fungal communities and the co‐occurrence networks in *S. breviflora* rhizosphere were more sensitive to long‐term grazing than bacteria. We reveal that rhizosphere effects and soil water content were the main drivers of the changes in fungal communities and their co‐occurrence networks. Moreover, the dominant bacterial phyla Bacteroidetes and Proteobacteria and fungal phyla Ascomycota and Glomeromycota might participate in regulating processes of *S. breviflora's* response to grazing. Overall, these findings give new snapshots of mechanisms of how grazing affects soil microbial communities, in an attempt to contribute to a clearer understanding of grazing‐tolerant mechanism of *S. breviflora*.

## Graphical abstract

In this study, we used a random matrix theory (RMT)‐based network approach to investigate the characterization of soil microbes associated with a grazing‐tolerant grass species, *Stipa breviflora*. I believe the results would provide new insight into the mechanisms by which grazing affects soil microbial communities, so as to contribute to a better understanding of grazing‐tolerant mechanism of *S. breviflora*.

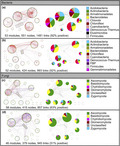

## 1 INTRODUCTION

The soil microbial community plays an important role in grassland ecosystem dynamics and has a crucial influence upon plant ecophysiological traits (Andres et al., 2017; Ford, Rousk, Garbutt, Jones, & Jones, 2013). A major challenge in applied ecology is to understand response mechanisms of those complex microbial communities to grazing, especially for those associated with grass species tolerant of grazing. Many studies have demonstrated that herbivores largely determine aboveground biomass, and also directly and indirectly affect the belowground soil microbial community through their impact on plants and soil properties (Dawson, Grayston, & Paterson, 2000; Yang et al., 2013). In this interaction, a common response of grazed grass plants to browsing is the stimulation of microbial processes and nitrogen availability within their rhizospheres, and the reallocation of belowground resources to aboveground structures (Bardgett, Wardle, & Yeates, 1998; Paterson, 2003). In response to defoliation, soil microbes either positively or negatively affect their host plant growth through nutrient transformation, phytohormone synthesis, and pathogen inhibition (Wardle, Bardgett, Klironomos, Setälä, & Van der Putten, 2004). Recently, an increasing number of researches have focused on the role of top‐down effects and bottom‐up feedback (Chen et al., 2018; Eldridge & Delgado‐Baquerizo, 2018). However, these researches have been mostly limited to single properties of soil microbial composition and their function (Andres et al., 2017; Ford et al., 2013), rather than explicitly considering the characteristics associated with a grazing‐tolerant grass species under extreme grazing stress.

In natural habitats, soil microbial community coexists in complex arrays and has a highly structured complex network (Faust & Raes, 2012). It has been shown that the response characteristics of soil microbial communities can be influenced by certain numerical properties of interaction networks under the environmental change (De Vries & Wallenstein, 2017). The recent emergence of microbial network analysis has revealed an array of astonishing potential interactions and strong linkages between taxa within soil microbial communities and uncovered ubiquitous characteristics of microbes in soils (Shi et al., 2016; Zhou et al., 2010; Zhou, Deng, Luo, He, & Yang, 2011). Such studies can provide insights into community composition and interactions among soil microbes at the community level that could not be obtained by traditional analytical approaches under grazing stress conditions (Zhang, Liu, Song, Wang, & Guo, 2018). Accordingly, the better understanding of the interaction networks of soil microbial communities, as well as the interdependent relationships among taxa under long‐term grazing, is critical for better understanding the grazing tolerance mechanisms of different host plants and for implementing restoration management and sustainable development programs in degraded grassland ecosystems (Newman, 2006; Shi et al., 2016).

*Stipa breviflora* is a palatable, grazing‐tolerant, and drought‐resistant grass species, which grows rapidly during springtime in desert steppe communities and is widely distributed in the western Inner Mongolia (Ren et al., 2017). Since this plant species plays a central role in soil and water conservation and desertification control (Zhang, Niu, Wu, Buyantuyev, & Dong, 2012), it has attracted attention for its potential use in the restoration of degraded grasslands. While vegetation and soil responses to grazing have been elucidated (Lu, Zhou, Wang, & Song, 2016), there is limited empirical description of the soil microbial community structure and composition hosted by *S. breviflora* (Gao, Han, & Zhang, 2017). Crucially, the potential interactions of soil microbial community members and mechanisms of *S. breviflora* grazing tolerance remain unclear.

In this study, we characterized soil microbes from the rhizosphere and bulk soil of a grazing‐tolerant grass species, *S. breviflora*. We aimed to address the following questions: (a) Are the soil microbes in the rhizosphere and bulk soil of *S. breviflora* different in response to long‐term grazing? and (b) How do different soil microbial taxa of grazing‐tolerant grass species, *S. breviflora*, interact with each other in response to long‐term grazing?

## 2 MATERIALS AND METHODS

### 2.1 Site description and sample collection

Sampling was conducted in the Siziwang Banner Research Station in western Inner Mongolia, China (41°47′17″–41°47′28″N, 111°54′07″–111°54′55″E, at 1,441 m elevation). The area has a temperate continental arid and semiarid climate, with a mean annual temperature ranging from 5.0 to 8.5 C and annual precipitation from 220 to 280 mm, 60%–80% of which falls from June to September. The main soil types in this area are light chestnut soil. The study site was divided into ungrazed and grazed areas of grassland, where the ungrazed grassland (500 m × 500 m) had been excluded from grazing for 13 years (from 2005 to 2017), and the grazed area was grazed by sheep during the whole growing season at a relatively high grazing intensity (>2 SE/ha, where SE represents 50 kg Sheep Equivalent) (Xu, Wang, Li, Wang, & Han, 2019). The daily grazing schedule was from 6:00 a.m. to 6:00 p.m. Vegetation within the ungrazed area was dominated by *S. breviflora*, *Cleistogenes squarrosa*, and *C. songorica*, with *Allium tenuissimum*, *Artemisia scoparia*, *A. frigida*, *Kochia prostrate*, *Convolvulus ammannii*, and *Caragana stenophylla*, and the open‐grazed area was dominated by *S. breviflora*, *Al. tenuissimum*, and *A. frigida*.

Samples were collected from five 5‐m × 5‐m plots, which were separated by 50 m, in the grazed and ungrazed grassland areas in August 2017. In each plot, ten *S. breviflora* plants and associated rhizosphere and bulk soil were carefully excavated. Rhizosphere soil was sampled from the soil that firmly adhered to the root; bulk soil was sampled from the soil that easily shaken from the roots. Rhizosphere and bulk soil samples were sieved to 2 mm and immediately stored at –80°C until DNA extraction.

The basic characteristics of bulk soils in the ungrazed plots comprised water content: 10.17%; pH: 8.34; total organic carbon (TOC): 14.49 g/kg; total N (TON): 1.20 g/kg; and total P: 0.27 g/kg. Basic characteristics of bulk soils in the grazed plots were water content: 6.77%; pH: 8.14; TOC: 12.00 g/kg; TON: 1.42 g/kg; and 0.34 g/kg of P.

### 2.2 Soil DNA extraction and quantitative PCR analyses

Total genome DNA was extracted from five replicates of 0.5 g of soil samples using a FastDNA SPIN Kit for soil (MP Biomedicals, Santa Ana, CA, USA), according to the manufacturer's instructions, and quantified using NanoDrop ND‐1000 (Thermo Fisher Scientific, Wilmington, Delaware, USA); DNA concentration was determined using fluorometric analysis with a microplate reader (SpectraMax M5, Molecular Devices, CA, USA) prior to storage at –80°C. Amplicons targeting the bacterial 16S rRNA genes of distinct regions (V3‐V4) were amplified using specific primers (341F: 5’‐CCTAYGGGRBGCASCAG‐3’ and 806R: 5’‐GGACTACNNGGGTATCTAAT‐3’) (Thijs et al., 2017), and the fungal ITS genes of distinct regions (ITS1‐ITS2) were amplified using specific primers (ITS1F: 5’‐CTTGGTCATTTAGAGGAAGTAA‐3’ and ITS2R: 5’‐GCTGCGTTCTTCATCG ATGC‐3’) (Orgiazzi et al., 2012). PCR reactions were carried out using a Phusion High‐Fidelity PCR Master Mix (New England Biolabs, Ipswich, MA, USA) in a 30 μL mixture that comprised 15 μL of Phusion High‐Fidelity PCR Master Mix (2×), 0.2 μM of forward and reverse primers, 10 ng of template DNA, and PCR‐grade water up to 30 μL. Thermal cycling consisted of initial denaturation at 98°C for 1 min, followed by 30 cycles of denaturation at 98°C for 10 s, annealing at 50°C for 30 s, and elongation at 72°C for 30 s, with a final extension at 72°C for 5 min. The obtained products were purified using a GeneJET Gel Extraction Kit (Thermo Fisher Scientific, Wilmington, Delaware, USA) and quantified using a Qubit 2.0 Fluorometer (Thermo Fisher Scientific, Wilmington, Delaware, USA) and Agilent Bioanalyzer 2100 (Agilent Technologies, Santa Clara, CA, USA) system. Finally, the library was sequenced on an Ion S5 XL (Thermo Fisher Scientific, Wilmington, Delaware, USA) platform, and 600‐bp single‐end reads were generated at the Novogene Bioinformatics Technology Co., Ltd., Beijing, China.

### 2.3 Sequence analysis

Single‐end reads were assigned to samples based on their unique barcode and truncated at the barcode and primer sequence. Quality filtering on the raw reads was performed under specific filtering conditions to obtain high‐quality clean reads, according to the Cutadapt v1.9.1 quality controlled process (Martin, 2011). Reads were compared with a reference database (Gold database) using the UCHIME algorithm to detect chimaera sequences, which were subsequently removed, and effective tags were obtained (Edgar, Haas, Clemente, Quince, & Knight, 2011). Sequences were analyzed using Uparse software (v7.0.1001) (Edgar, 2013), where sequences with ≥97% similarity were assigned to the same OTUs (operational taxonomic units). For each representative sequence, taxonomic information for bacteria was annotated using the Silva Database (https://www.arb‐silva.de/) based on an RDP classifier (v2.2) algorithm, and taxonomic information for fungi was annotated using the Unite Database (https://unite.ut.ee/) based on the Blast algorithm that was calculated in QIIME software (v1.9.1) (Koljalg et al., 2013). Phylogenetic relationships of OTUs and differences among dominant species in samples (groups) were analyzed using multiple sequence alignments in MUSCLE software (v3.8.31) (Edgar, 2004).

### 2.4 Network analysis

We analyzed bacterial and fungal networks, based on OTU relative abundance, to understand interactions and responses to grazing treatment. Networks were constructed using molecular ecological network analyses (MENA) (http://ieg4.rccc.ou.edu/mena) based on random matrix theory (RMT) algorithms (Deng et al., 2012), and visualized using Cytoscape v3.7.1 (Shannon et al., 2003). Thresholds in the network construction were automatically chosen, and module separation was based on the fast greedy modularity optimization (Deng et al., 2012; Zhou et al., 2010). The modularity (*M*) index measures the extent to which a network is divided into modules; we used *M* > 0.4 to define modular structures (Newman, 2006). Keystone taxa, including module hubs and connectors, were identified using within‐module connectivity (*Zi*) and among‐module connectivity (*Pi*) values, where module hubs (highly connected to many nodes within modules) were *Zi* > 2.5 and *Pi ≤ *0.62; connectors (highly linked to several modules) were *Zi ≤ *2.5 and *Pi* > 0.62; peripheral nodes (nodes connected in modules with few outside connections) were *Zi ≤ *2.5 and *Pi ≤ *0.62; and network hubs (highly connected nodes within entire network) were *Zi* > 2.5 and *Pi* > 0.62 (Zhou et al., 2011). Random networks were constructed to compare with the original network to determine general network characteristics (Zhou et al., 2011). For each identified network, we generated 100 randomly rewired networks, and calculated all network indexes individually (Maslov & Sneppen, 2002).

### 2.5 Statistical analysis

Soil bacterial and fungal alpha diversity was calculated using Shannon index, and the richness was calculated using Chaol index in QIIME (Version1.9.1) (Caporaso et al., 2010), and the differences were tested using one‐way analysis of variation (ANOVA) followed by Tukey's honestly significant difference test (at *p* < .05) in SPSS 21.0. We used nonmetric multidimensional scaling (NMDS) analysis on the basis of Bray–Curtis similarity distances in R (Version 3.5.2) to analyze similarities of bacterial and fungal community composition (Kuczynski et al., 2011). Differences between grazed and ungrazed grassland bacterial and fungal community composition were tested using analysis of similarities (ANOSIM) at* p* < .05, and Student's *t* test was used to search for statistically different biomarkers between grazed areas and ungrazed areas, based on *p* < .05.

## 3 RESULTS

### 3.1 Soil microbial diversity

**FIGURE 1:**
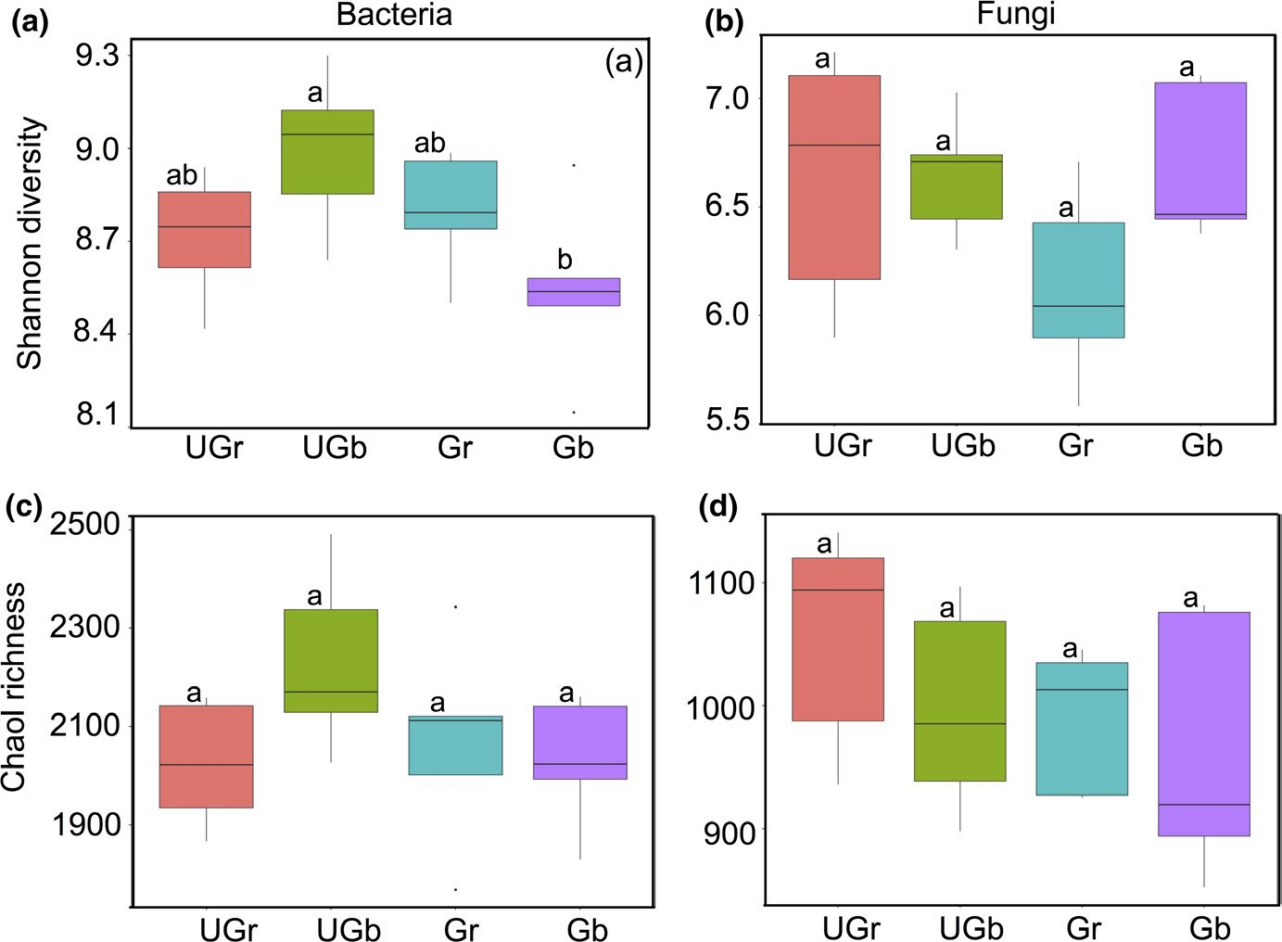
Bacterial and fungal diversity and richness index in grazed and ungrazed grassland soils. Data means ± SE (*n* = 5). Significant differences (*p* < .05) between different treatments are labeled with different letters (Tukey's honestly significant difference test). Gb, bulk soil of grazed plots; Gr, rhizosphere soil of grazed plots; UGb, bulk soil of ungrazed plots; UGr, rhizosphere soil of ungrazed plots

A total of 709,058 high‐quality sequences clustered into 3,816 bacterial OTUs and 787,833 high‐quality sequences clustered into 2,991 fungal OTUs. The Shannon diversity indices showed that bulk soil bacterial communities were significantly more diverse in ungrazed grassland plots than those of grazed grassland plots (*p* < .05; Figure 1a). However, no differences in the richness of bacteria and fungi were found between all samples (*p* > .05; Figure 1c, d).

### 3.2 Soil microbial community composition

**FIGURE 2:**
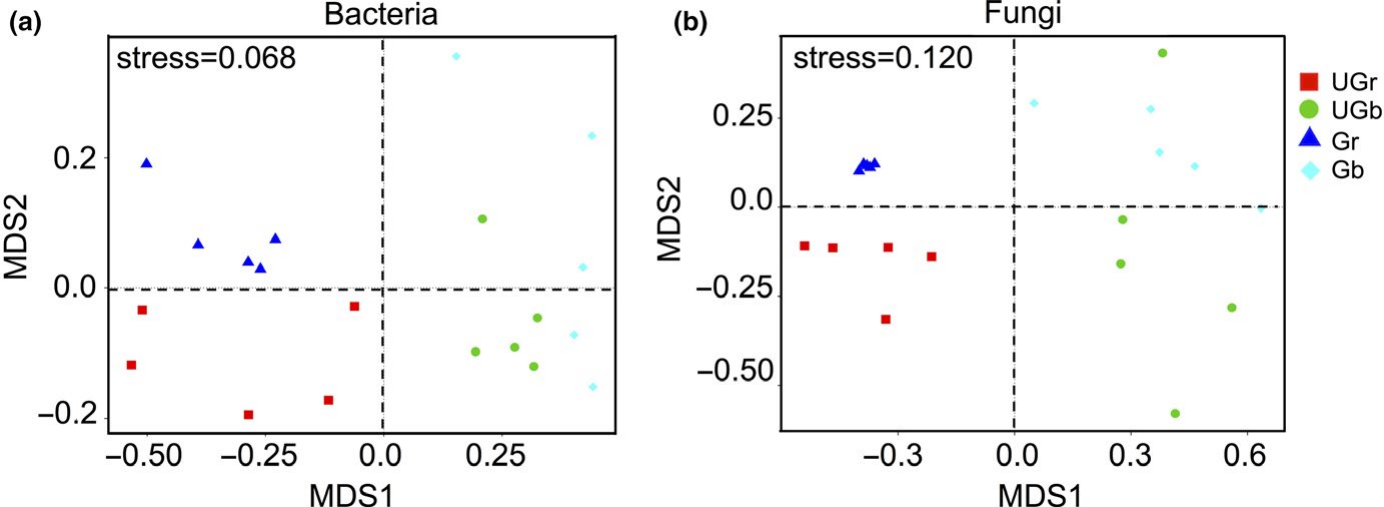
NMDS plots of the bacterial (a) and fungal (b) communities in the grazed and ungrazed grassland soils. A stress level of <0.20 was considered acceptable for NMDS plots. Gb, bulk soil of grazed plots; Gr, rhizosphere soil of grazed plots; UGb, bulk soil of ungrazed plots; UGr, rhizosphere soil of ungrazed plots

Bacterial and fungal community composition varied according to site (grazed and ungrazed plots) and soil compartments (rhizosphere or bulk soil) (stress < 0.2; Figure 2). ANOSIM pairwise comparisons showed that grazing treatment significantly distinguished between bacterial and fungal communities in rhizosphere (*p* = .009 and *p* = .008, respectively), but not in bulk soil (*p* = .379 and *p* = .053, respectively).

### 3.3 Soil microbial interaction networks

The values of the average clustering coefficient (avgCC), harmonic geodesic distance (HD), path distance (GD), and *M* values of all networks were larger than those for randomized networks, indicating the RMT‐based network approach was effective for the identification of soil microbe–microbe interactions (Table 1).

**TABLE 1:** Topological properties of the bacterial and fungal networks in grazed and ungrazed grassland soils

Topological characteristics	16S		ITS	
	Ungrazed area	Grazed area	Ungrazed area	Grazed area
Commonly present OTU No.	1961	1554	621	593
Empirical network				
Total nodes	551	424	415	379
Total links	1,483	993	857	945
Links per node	2.69	2.34	2.07	2.49
*R* ^2^ of power law	0.872	0.881	0.902	0.904
Similarity threshold	0.960	0.950	0.870	0.880
avgK	5.383	4.684	4.130	4.987
avgCC	0.186	0.182	0.164	0.199
HD	4.127	4.444	4.321	3.902
GD	4.993	5.608	5.442	4.782
Modularity	0.630	0.653	0.592	0.525
Random networks^a^				
avgCC ± *SD*	0.047 ± 0.005	0.034 ± 0.005	0.037 ± 0.005	0.043 ± 0.006
HD ± *SD*	3.223 ± 0.022	3.330 ± 0.031	3.425 ± 0.031	3.232 ± 0.032
GD ± *SD*	3.525 ± 0.032	3.681 ± 0.043	3.787 ± 0.046	3.573 ± 0.046
Modularity ± *SD*	0.391 ± 0.004	0.430 ± 0.005	0.470 ± 0.005	0.408 ± 0.005

Abbreviations: avgK, average degree; avgCC, average clustering coefficient; HD, average harmonic geodesic distance; GD, average path distances.

^a^ Random networks were generated by rewiring all of the links of a corresponding empirical network with the identical nodes and links. Data were generated from 100 random runs, and *SD* indicates the standard deviation from the 100 runs.

**FIGURE 3:**
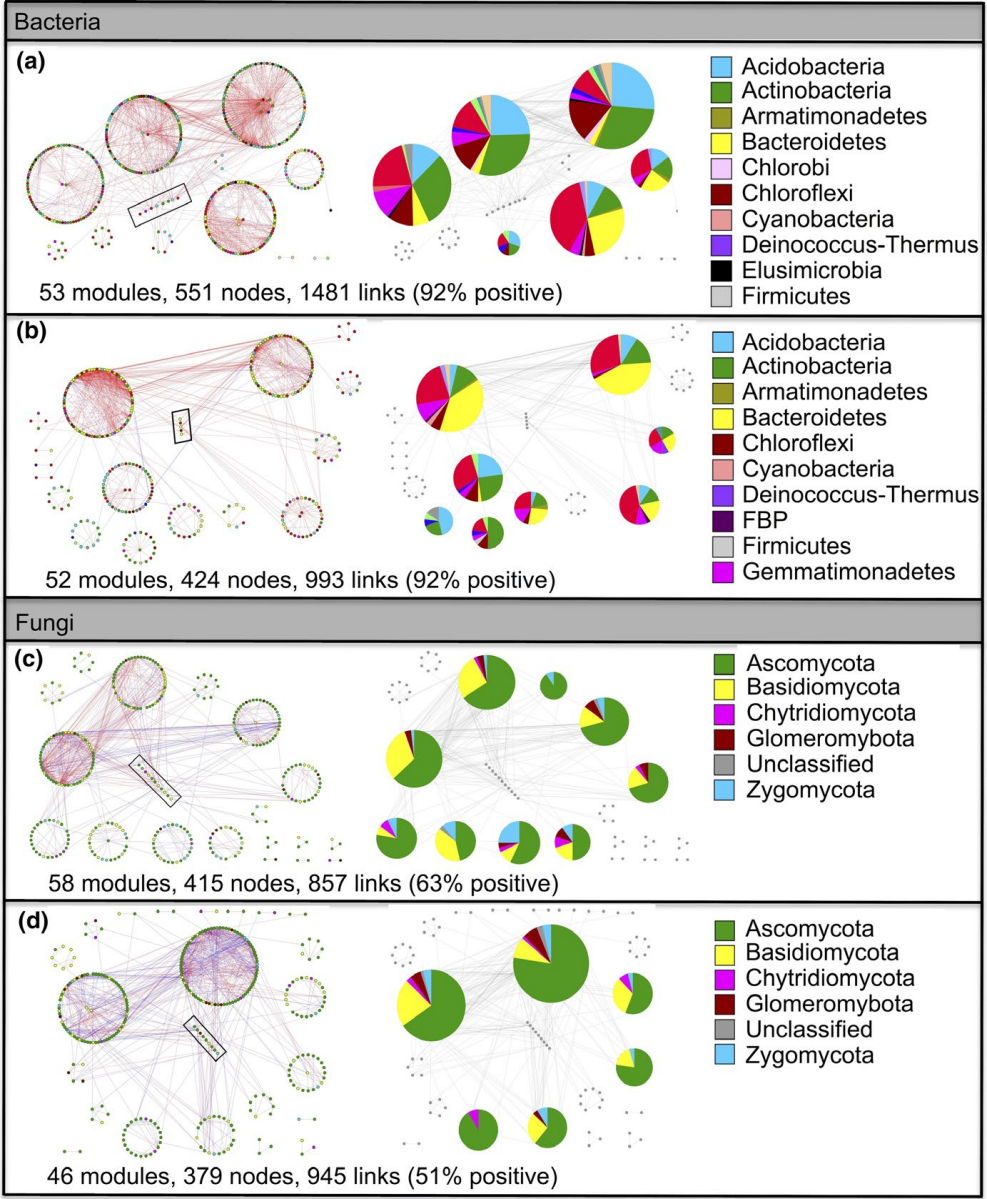
Highly connected modules of bacteria and fungi in grazed and ungrazed grassland soils. Colors of nodes indicate different major phyla; pie charts represent the composition of modules with >10 nodes. A red link indicates a positive correlation between two individual nodes, whereas a blue link indicates a negative correlation. Nodes at module centers are module hubs, and nodes in black boxes are connectors

Bacterial networks were smaller and less connected in grazed soils than in ungrazed soils, with 23.05% less nodes and 32.95% less links in grazed grassland soils (Figure 3b), whereas fungal networks were smaller but more connected in grazed soils than in ungrazed soils, with 8.67% less nodes and 10.27% more links in grazed grassland soils (Figure 3d). Grazing slightly decreased positive links in the fungal networks (Figure 3d).

### 3.4 Keystone taxa

**FIGURE 4:**
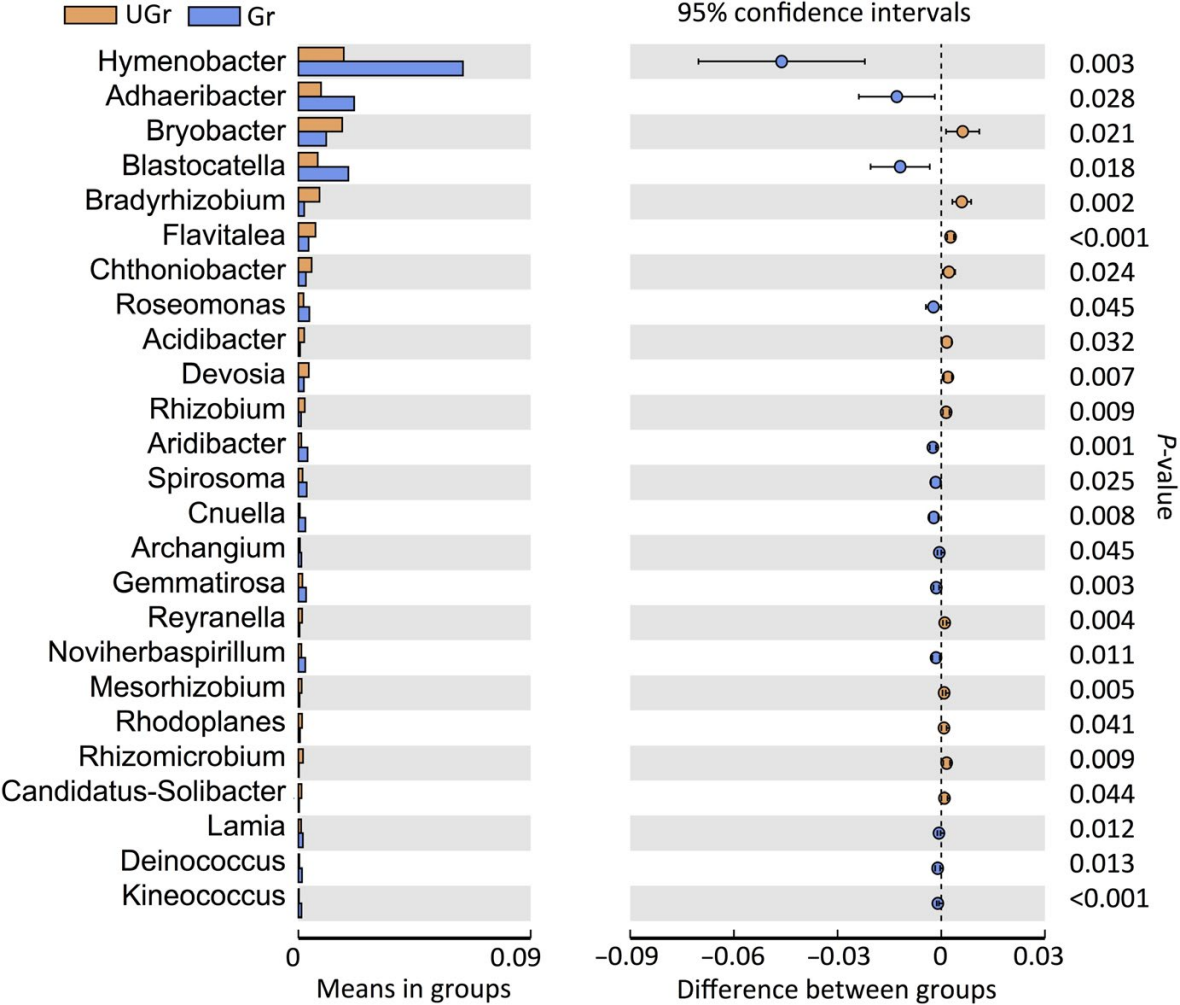
Relative abundances of bacterial genera that showed significant differences (*p* < .05) between grazed and ungrazed grassland. Student's *t* test was used to evaluate the significance of differences between treatments. Gr, rhizosphere soil of grazed plots; UGr, rhizosphere soil of ungrazed plots

**FIGURE 5:**
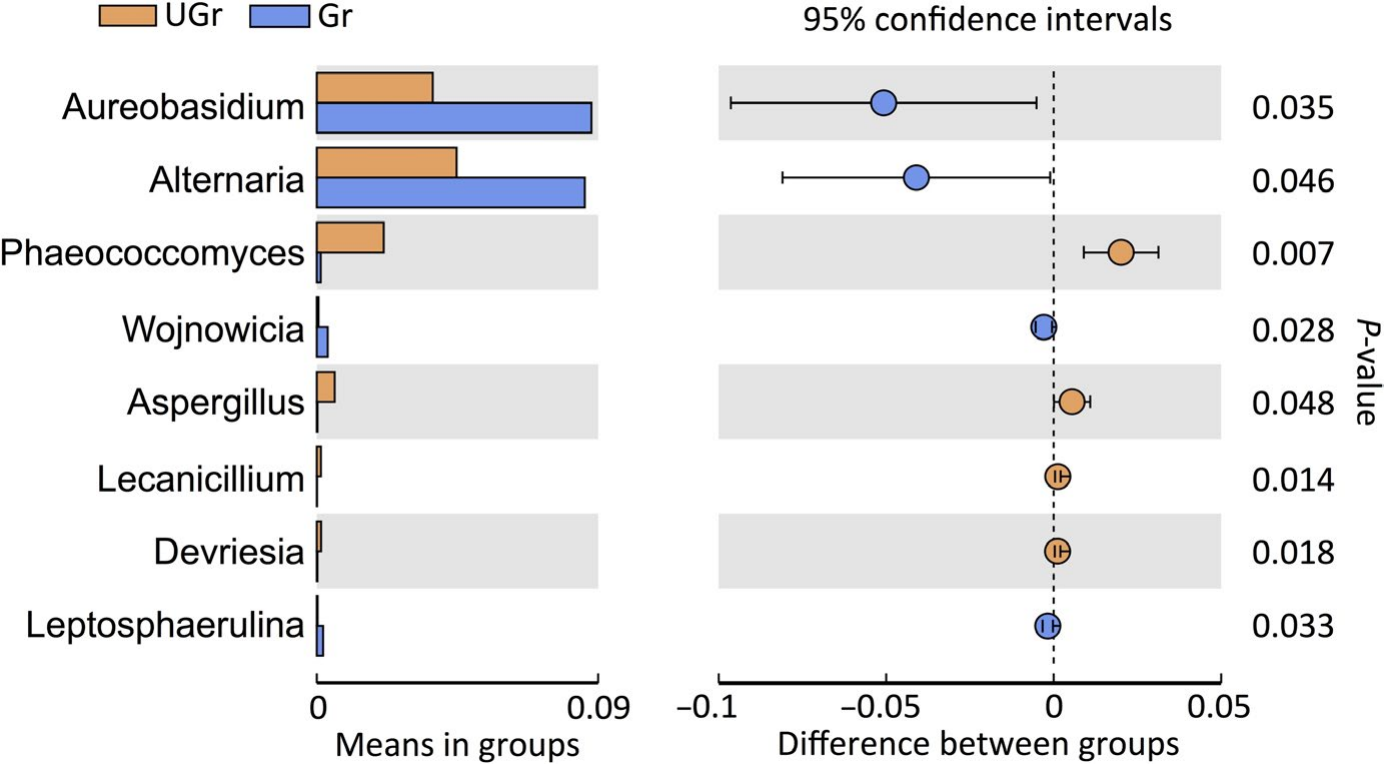
Relative abundances of fungal genera that showed significant differences (*p* < .05) between grazed and ungrazed grassland. Student's *t* test was used to evaluate the significance of differences between treatments. Gr, rhizosphere soil of grazed polts; UGr, rhizosphere soil of ungrazed plots

Genus‐level relative abundance data showed that thirteen bacterial indicators and four fungal indicators were increased in the rhizosphere of grazing area. *Hymenobacter*, *Adhaeribacter*, and *Blastocatella* were the most common bacterial genera in the rhizosphere (Figure 4), whereas *Aureobasidium* and *Alternaria* were most abundant in the rhizosphere under grazing condition (Figure 5).

According to *Zi* and *Pi* values, we recorded eight module hubs and five connectors in the bacterial networks of grazed grassland soils, which mainly derived from phyla Bacteroidetes and Proteobacteria in the rhizosphere, whereas three module hubs and nine connectors were recorded in the fungal networks of grazed grassland soils. These connectors and module hubs mainly derived from phyla Ascomycota and Basidiomycota in the bulk soil (Table 2).

**TABLE 2:** Classification of module hubs and connectors from bacterial and fungal networks in grazed grassland soils. The order: phylum, class, order, family, and genus classification

	OUT No.	*Zi*	*Pi*	Links	Lineage
Bacteria					
Connectors	OTU_80	0.960	0.640	15	Bacteroidetes, Sphingobacteriia, Sphingobacteriales, Chitinophagaceae, Segetibacter
	OTU_333	−0.867	0.625	4	Bacteroidetes, Sphingobacteriia, Sphingobacteriales, Chitinophagaceae, Flavisolibacter
	OTU_661	−1.237	0.640	5	Bacteroidetes, Sphingobacteriia, Sphingobacteriales, Chitinophagaceae, Segetibacter
	OTU_295	−0.852	0.625	4	Proteobacteria, Alphaproteobacteria, Rhodospirillales, Geminicoccus
	OTU_915	0.353	0.625	4	Chloroflexi, TK10
Module hubs	OTU_98	2.705	0	25	Bacteroidetes, Sphingobacteriia, Sphingobacteriales, Chitinophagaceae, Cnuella
	OTU_148	2.834	0.153	13	Proteobacteria, Alphaproteobacteria, Sphingomonadales, Sphingomonadaceae
	OTU_271	2.834	0	11	Proteobacteria, Alphaproteobacteria, Rhodobacterales, Rhodobacteraceae, Rubellimicrobium
	OTU_451	4.032	0.133	14	Actinobacteria, MB‐A2−108
	OTU_3783	3.176	0	8	Actinobacteria, MB‐A2−108
	OTU_93	3.255	0	9	Proteobacteria, Betaproteobacteria, Burkholderiales, Alcaligenaceae
	OTU_388	2.696	0.197	9	Proteobacteria, Deltaproteobacteria, Desulfurellales, Desulfurellaceae, H16
	OTU_1252	2.828	0	16	Proteobacteria, Deltaproteobacteria, Myxococcales, Haliangiaceae, Haliangium
Fungi					
Connectors	OTU_2968	−0.763	0.625	2	Ascomycota, Lecanoromycetes, Lecanorales, Parmeliaceae, Cetraria
	OTU_447	0.675	0.700	7	Basidiomycota, Agaricomycetes
	OTU_179	0.028	0.656	2	Zygomycota, unclassified Zygomycota, Mortierellales, Mortierellaceae
	OTU_25	1.325	0.651	8	Ascomycota, Eurotiomycetes, Eurotiales, Trichocomaceae
	OTU_1424	−0.502	0.667	3	Ascomycota, Eurotiomycetes, Eurotiales, Trichocomaceae
	OTU_100	1.225	0.656	5	Ascomycota, Pezizomycetes, Pezizales, Pyronemataceae
	OTU_233	0.283	0.625	3	Ascomycota, Sordariomycetes, Hypocreales, Ophiocordycipitaceae, Hirsutella
	OTU_277	−0.798	0.625	3	Ascomycota, Sordariomycetes, Hypocreales, Nectriaceae
	OTU_913	−0.619	0.625	1	Glomeromycota, Glomeromycetes, Glomerales, Glomeraceae
Module hubs	OTU_5	2.890	0.266	9	Basidiomycota
	OTU_26	3.493	0.430	2	Basidiomycota, Agaricomycetes, Cantharellales
	OTU_34	3.673	0.287	14	Basidiomycota, Tremellomycetes, Tremellales, Incertae_sedis_Tremellales, Dioszegia

Note

Modules hubs have *Zi* > 2.5 and *Pi* ≤ 0.62, whereas connectors have *Zi* ≤ 2.5 and *Pi* > 0.62.

## 4 DISCUSSION

### 4.1 Shifts of soil microbial community composition

This study focused on the identification of characterization of soil microbes associated with a grazing‐tolerant grass species, *S*. *breviflora*. We found that long‐term grazing greatly affected rhizosphere microbial community composition, but did not influence them in the bulk soil, indicating that the influence of grazing was more evident in structuring the microbial communities present in rhizosphere than bulk soils (Hamilton & Frank, 2001). These results are consistent with previous findings that grazing has a significant impact on the rhizosphere bacterial community structure, but has no effect on bulk soil bacterial community structure in *S*. *breviflora* desert steppe (Zhang et al., 2019). Evidence suggests that effects of grazing on soil microbes have been attributed to increased release of root exudates that are rapidly used by rhizosphere microbes and increase available N in plant shoots; this process may result in a positive feedback to the plant of improved nutrient recycling and uptake (Bardgett et al., 1998). It has also been shown that grazing‐tolerant grass species responded to defoliation by increasing the allocation of resources to aboveground tissue and reduction in root biomass (Guitian & Bardgett, 2000). These findings indicated that reduction in root C allocation leads to lower amount of resources to belowground, which ultimately affected the soil microbial community (Bardgett et al., 1998). Consequently, we proposed that shift of microbial community composition was presumably related to the exudative response of *S. breviflora* to defoliation (Paterson, 2003).

In addition, soil properties have been considered as the crucial drivers of the changes in the composition of soil microbial communities (Calleja‐Cervantes et al., 2015). We found that grazing increased TN and TP and decreased soil water content, TOC, and pH. This finding was in general agreement with previous studies that were conducted in different grassland types (Andres et al., 2017; Yang et al., 2013). Previous studies showed that grazing could increase N inputs into the microbial community and ultimately increases the soil microbial activity (Guitian & Bardgett, 2000; Yang et al., 2013). This increase in soil microbial activity could feedback to the plants by increasing soil nutrient availability, which supports more greater shoot nutrient to rapid regrowth of aboveground tissue following defoliation (Cantarel et al., 2017). However, numerous studies have concluded that long‐term grazing will decrease soil water content and TOC through soil trampling and the removal of vegetation, which would also negatively affect soil microbial communities (Eldridge & Delgado‐Baquerizo, 2018; Zhang, Liu, et al., 2018). Our findings supported these observations. Therefore, we believe that soil properties also play an important role in shaping microbial communities, ultimately driving changes in plant community composition.

### 4.2 Characterization of soil microbial interactions

Our results showed that grazing affected the co‐occurrence networks of overall fungal networks more than those of bacterial networks. Fungal networks in grazed grassland soils had stronger interaction, higher average degree, shorter path distance, lower modularity, and more negative correlations than ungrazed grassland soils, suggesting the more sensitive and quick response to environmental changes under long‐term grazing (De Vries et al., 2018). On the contrary, bacterial networks in grazed grassland soils had more cooperative relationships within bacterial members, indicating higher stability and more tolerance for stress imposed by grazing disturbance (Zhang, Zhang, Liu, Shi, & Wei, 2018). This may be due to a direct effect of grazing by reducing the soil carbon substrates available for colonization. Fungi are more dependent than bacteria on soil carbon substrates (Deng et al., 2012), which result in a higher level of competition for a reduced supply of nutrients from plants and soil in the grazing grassland (Chen et al., 2018). Studies have found that heavy grazing decreased organic carbon stock in soil through decreasing productivity of plant biomass, roots, and litter in *S. breviflora* desert steppe (Wang et al., 2017). Therefore, the reduction of TOC increased the competition for soil carbon substrate within fungal community, which possibly weakened the microbial associations and consequently could decrease the system stability to resist to adverse environmental conditions (Newman, 2006). Interestingly, we found that more rhizosphere communities were regarded as connectors and module hubs in bacterial co‐occurrence network under grazing treatment, while more bulk soil communities dominated in fungal co‐occurrence network under grazing treatment. This may be due to the fact that bacterial community develop faster than fungal communities and can use root exudates more quickly (Paterson, 2003), while fungi may not have many available niches in the rhizosphere (Shi et al., 2016). These findings are consistent with the result that bacteria are the most abundant microbes in the rhizosphere due to their excellent substrate utilization capacities (Dawson et al., 2000). A recent research showed that grazing increased the dominant role of *S. breviflora* in the plant communities, which would have occupied more niche space in the community that thereby reduced the number of coexisting species possible (Lv et al., 2020). This increase in dominant role also indicated that *S. breviflora* exert selective forces to influence microbial community in rhizosphere, thereby resulting in strong interspecific competition within microbial communities (Godoy, Bartomeus, Rohr, & Saavedra, 2018). Therefore, we proposed that the rhizosphere effects of *S. breviflora* presumably modified the soil microbial interactions following herbivory (De Vries & Wallenstein, 2017).

### 4.3 Keystone taxa response to grazing

The links between plant species and soil microbes are long established (Cantarel et al., 2017), but understanding the relationship between grazing‐tolerant plant species and soil microbes was previously unclear. An additional goal of this study was to determine soil bacterial and fungal keystone taxa, which would be beneficial for host plant adaptation to grazing. We identified several system‐specific taxa in the *S*. *breviflora* rhizosphere in grazed grassland soils. For example, the phyla Bacteroidetes and Proteobacteria, as the most dominant bacteria, were the strongest responders to grazing and drive the bacterial network structure, indicated that *S*. *breviflora* established root‐inhabiting microbial communities by selecting a limited number of phyla (Dawson et al., 2000). *Hymenobacter*, the most dominant bacterial genus in the rhizosphere, has been reported as a plant growth‐promoting bacteria, could increase the antioxidative properties and content of fatty acids and phenolic compounds in plants (Dimitrijevic et al., 2018). *Adhaeribacter* has the same lineage as *Hymenobacter*, which could indicate a genus that specializes in the degradation of complex products derived from different composts (Calleja‐Cervantes et al., 2015). *Blastocatella* has been reported elsewhere to be associated with phosphorus‐accumulating herb, played key roles in mobilizing soil mineral‐P (Ye et al., 2020). In particular, co‐occurrence network analyses identified several genera (e.g., *Cnuella*, *Flavisolibacter*, and *Segetibacter*) of the family Chitinophagaceae, which was a type of ammonia‐oxidizing bacteria (AOB). These specific taxa identified above could play important roles in nitrogen transformation (Wu et al., 2019). Other keystone taxa including family Sphingomonadaceae (oligotrophic character), family Alcaligenaceae (related to phenolic compounds), family Desulfurellaceae (sulfate‐reducing bacteria), and genus *Haliangium* (halophilic myxobacteria) are related to the transformations of complex product (Calleja‐Cervantes et al., 2015; Dimitrijevic et al., 2018). These observed taxa might play important roles in maintaining cooperative associations within bacterial community to survive under grazing stress (Zhang, Zhang, et al., 2018). Although little is known about their grazing tolerance mechanisms, however, their ability to maintain network stability might suggest that these taxa adapted to the long‐term grazing and might play an important role in the *S*. *breviflora* rhizosphere.

In addition, *Aureobasidium* and *Alternaria* were the most dominant fungal genera in the *S*. *breviflora* rhizosphere, which belong to phylum Ascomycota, indicating that Ascomycota dominated the composition of root‐associated fungi, which is in line with the results of previous studies (Chen et al., 2018; Yin, Nan, Li, & Hou, 2008). *Aureobasidium* is an endophytic or epiphytic fungus that is beneficial to its host plant and helps the plant endure heavy metal ions, osmotic pressure, and extreme environmental conditions (Chi et al., 2009). When a greater abundance of this genus occurs in the *S*. *breviflora* rhizosphere, it would positively feedback into host plant, so as to response to grazing stress (Bardgett et al., 1998; Wardle et al., 2004). Previous study has reported that fungal drought‐tolerant taxa mainly belong to phyla Ascomycota and Glomeromycota, whereas drought‐sensitive taxa mainly belong to phylum zygomycota (family Mortierellaceae) (De Vries et al., 2018). In the present study, we identified several drought‐tolerant taxa Pyronemataceae (phylum Ascomycota) and Glomeraceae (phylum Glomeromycota), and drought‐sensitive taxa Mortierellaceae (phylum zygomycota), indicating that SWC also drive the fungal interactions. Grazing decreased the total aboveground biomass, resulting in decreased SWC, which indirectly affected the soil fungal network, in this case, triggering strong interspecific competition, potentially weakening network stability. This assumption would explain why fungal networks show more sensitive to grazing than bacterial networks in the *S. breviflora* desert steppe. In contrast, *Alternaria* is a common saprophyte, majority of species are animal and plant pathogens, and often occurring in plant leaf spot and tissue decay (Liu, Li, Hu, Wang, & Gao, 2018). Recent study has shown that the accumulation of root pathogens can produce negative feedback to plant growth through directly reducing root uptake capacity (De Vries & Wallenstein, 2017). However, other studies have shown that the root pathogens do not cause the same level of negative feedback to all plant species in the community, and thus, their existence can lead to the qualitative difference in plant community composition (Wardle et al., 2004). Therefore, we cannot definitively conclude whether these taxa are beneficial for *S. breviflora* adaptation to grazing, but the presence of these keystone taxa might participate in regulating processes of *S. breviflora's* response to grazing stress.

## 5 CONCLUSIONS

The bacterial and fungal communities have different response characteristics to long‐term grazing, with fungal community was more sensitive to grazing than the bacterial community. Long‐term grazing greatly affected rhizosphere microbial communities, but did not influence them in the bulk soil. More importantly, the dominant bacterial taxa such as *Adhaeribacter*, Alcaligenaceae,* Blastocatella*, Chitinophagaceae, Desulfurellaceae,* Haliangium*, *Hymenobacter*, and Sphingomonadaceae and fungal taxa *Alternaria*, *Aureobasidium*, Glomeraceae, Mortierellaceae, and Pyronemataceae shed new light on the involvement of soil microbes in grazing. Further studies of the regulatory mechanisms of these taxa are essential to better understand the grazing‐tolerant characteristics of *S. breviflora* and enrich theoretical knowledge of plant–soil–microbe interactions under grazing conditions.

## CONFLICT OF INTEREST

The authors declare that they have no competing interests.

## AUTHOR CONTRIBUTION

**Lingling Chen:** Data curation (equal); Funding acquisition (equal); Writing‐original draft (equal); Writing‐review & editing (equal). **Yala Saixi:** Data curation (equal); Writing‐review & editing (equal). **Ru Yi:** Data curation (equal); Software (equal). **Taogetao Baoyin:** Conceptualization (equal); Funding acquisition (equal); Writing‐review & editing (equal).

## Data Availability

The bacterial and fungal raw sequence data from this study were deposited in the NCBI Sequence Read Archive (accession numbers were PRJNA627738).
